# Tazemetostat in relapsed/refractory follicular lymphoma: a propensity score–matched analysis of E7438-G000-101 trial outcomes

**DOI:** 10.18632/oncotarget.28229

**Published:** 2022-05-11

**Authors:** David G. Proudman, Deepshekhar Gupta, Dave Nellesen, Jay Yang, Beth A. Kamp, Khalid Mamlouk, Bruce D. Cheson

**Affiliations:** ^1^Analysis Group, Inc., Menlo Park, CA 94025, USA; ^2^Epizyme, Inc., Cambridge, MA 02139, USA; ^3^Lymphoma Research Foundation, New York, NY 10005, USA

**Keywords:** tazemetostat, propensity score matching, wild-type EZH2, follicular lymphoma, objective response rate

## Abstract

Purpose: In the tazemetostat E7438-G000-101 trial of relapsed/refractory (R/R) follicular lymphoma (FL), apparent superior efficacy was suggested for mutant-type (MT) *EZH2* versus wild-type (WT) status. However, clinical disparities might have contributed to this conclusion. This study aimed to estimate outcomes after minimizing differences in baseline characteristics.

Methods: Propensity scores for each participant with WT (*n* = 54) and MT (*n* = 45) status were generated based on the likelihood of being selected given their baseline characteristics. Participants were matched using a 1:1 nearest-neighbor approach.

Results: The propensity-matched sample included 56 participants (28 WT, 28 MT). Objective response rates (95% confidence interval [CI]) were 35% (22–48) in WT and 69% (55–83) in MT prior to matching and 50% (31–69) in WT and 71% (54–88) in MT after matching. Median progression-free survival values (95% CI) were 11.1 (5.4–16.7) in WT and 13.8 months (11.1–22.1) in MT prior to matching and 14.3 (11.1–∞]) and 14.8 months (10.7–∞]) in WT and MT matched groups, respectively.

Conclusions: This analysis suggests that efficacy outcomes for tazemetostat observed in participants with WT *EZH2* R/R FL may have been similar to those in participants with MT had the 2 cohorts been more closely matched.

## INTRODUCTION

Follicular lymphoma (FL) is typically a slow-growing or indolent form of non-Hodgkin lymphoma (NHL) that arises from B lymphocytes. It comprises 20% of all NHLs and 70% of the indolent lymphomas reported in American and European clinical trials [1]. From 2012 to 2016, the incidence of FL in the United States was 2.7 per 100,000 [2]. Tazemetostat, a first-in-class oral enhancer of zeste homolog 2 (EZH2) inhibitor, was approved by the US Food and Drug Administration (FDA) in June 2020 for adult patients with relapsed or refractory (R/R) FL with mutant (MT) *EZH2*, as detected by an FDA-approved test, and who have received at least 2 prior systemic therapies and for adult patients with R/R FL who have no appropriate treatment alternatives, regardless of their *EZH2* status [3].

The efficacy and safety of tazemetostat have been evaluated in an open-label, single-arm phase 2 trial (E7438-G000-101) [4]. Individuals of both *EZH2* mutation types—MT and wild type (WT)—were included in the trial. Both MT and WT showed response, with the objective response rates (ORRs) higher in the MT (69%) versus the WT group (35%). Lack of random assignment of MT and WT status led to meaningful differences in baseline population characteristics; thus, outcomes from the WT and MT cohorts cannot be directly compared using trial data. Poorer prognostics factors were observed in the baseline characteristics of those in the WT compared with the MT population. Matching analysis was conducted to estimate adjusted treatment effects of tazemetostat while minimizing a range of baseline patient-level characteristics. This analysis was considered important because the lower response rate observed in the WT cohort during the trial may have partly been an artifact of the differences in baseline populations.

## RESULTS

### Balance diagnostics

**Figure 1:**
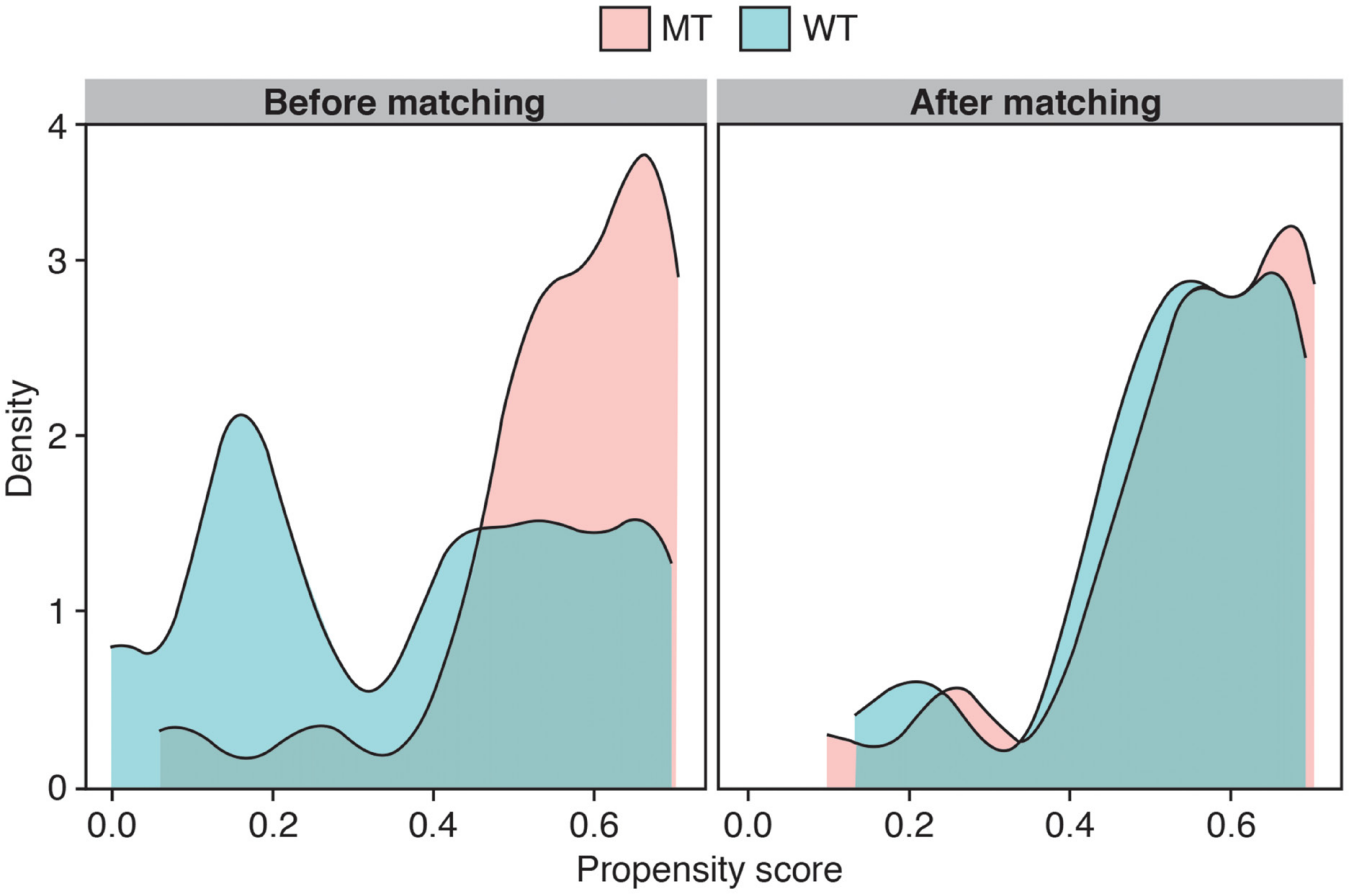
Propensity score density plots before and after matching. Abbreviations: MT: mutant type; WT: wild type.

The propensity-matched model produced a matched sample of 28 participants with *EZH2* MT and 28 with *EZH2* WT for an outcome comparison. The model estimated a Hosmer–Lemeshow goodness-of-fit test with *P* < 0.191, indicating the model is a reasonable fit with the data. (*P* > 0.05 indicates that the predicted probabilities of the outcome do not differ from the observed probabilities in a way that the binomial distribution cannot predict.) The MT and WT subpopulations were more comparable after matching according to propensity score, as evidenced by strong overlap of scores on a density plot (Figure 1).

In addition, standardized differences of all key baseline covariates selected for the propensity-estimation model were more similar between the WT- and MT-matched sample groups than between the WT and MT groups before matching (Table 1). Standardized differences between the MT and WT cohorts after matching also showed considerable reductions across other covariates such as number of prior lines of systemic anticancer therapy and number of participants with double refractory status. Among nonmatched variables, standardized differences were in some cases lower (eg, refractory status variables) and differences increased for some others (eg, age). Overall, these different measures provide evidence of a more balanced and comparable sample of MT and WT participants resulting from the matching procedure.

**Table 1:** Variables before and after matching

Variable	Before matching					After matching				
	Cohort group^a^		Mean difference (MT−WT)	Standardized mean difference	*P* value	Cohort group^a^		Mean difference (MT−WT)	Standardized mean difference	*P* value
	WT (*n* = 54)	MT (*n* = 45)				WT (*n* = 28)	MT (*n* = 28)			
**Matched**										
ECOG PS										
0	26 (48.2)	21 (46.7)	−1.5			16 (57.1)	15 (53.6)	−3.6		
1	23 (42.6)	24 (53.3)	10.7			12 (42.9)	13 (46.4)	3.6		
2	4 (7.4)	0 (0)	−7.4			0 (0)	0 (0)	0		
Unknown	1 (1.9)	0 (0)	−1.9	0.47	0.18	0 (0)	0 (0)	0	0.07	1.00
POD24	32 (59.3)	19 (42.2)	−17.0	0.35	0.14	14 (50.0)	12 (42.9)	−7.1	0.14	0.79
Prior ASCT	20 (37.0)	4 (8.9)	−28.2	0.71	<0.01	3 (10.7)	3 (10.7)	0	0	1.00
Line of anticancer therapy, *n*	3.7 ± 1.7	3.0 ± 1.7	−0.7 ± 0.3	0.40	0.05	3.1 ± 1.2	2.8 ± 1.4	−0.3 ± 0.4	0.25	0.36
Double refractory	15 (27.8)	9 (20.0)	−7.8	0.18	0.51	6 (21.4)	8 (28.6)	7.1	0.17	0.76
**Nonmatched**										
Age, mean ± SD, y	61.1 ± 11.4	61.8 ± 9.0	0.8 ± 2.1	0.08	0.71	64.9 ± 9.8	61.0 ± 9.2	−4.0 ± 2.5	0.42	0.13
Female sex	20 (37.0)	26 (57.8)	20.7	0.43	0.06	10 (35.7)	17 (60.7)	25.0	0.52	0.11
Grade 3b and transformed FL	6 (11.1)	3 (6.7)	−4.4	0.16	0.51	4 (14.3)	2 (7.1)	−7.1	0.23	0.67
Refractory to rituximab	32 (59.3)	22 (48.9)	−10.4	0.21	0.41	16 (57.1)	14 (50.0)	−7.1	0.14	0.79
Refractory to last therapy	22 (40.7)	22 (48.9)	8.2	0.16	0.54	11 (39.3)	12 (42.9)	3.6	0.07	1.00

^a^All are *n* (%) unless otherwise indicated. Abbreviations: ASCT: autologous stem cell transplantation; ECOG PS: Eastern Cooperative Oncology Group performance status; FL: follicular lymphoma; MT: mutant type; POD24: progression of disease ≤24 mo; SD: standard deviation; WT: wild type.

### Propensity score–matched outcomes

Efficacy results in the form of ORR were compared before and after matching for the *EZH2* MT and WT cohorts (Table 2). The point estimate ORR was more similar for the matched sample (MT, 71%; WT, 50%) compared with the sample before matching (MT, 69%; WT, 35%).

**Table 2:** Objective response rates before matching

Population	Before matching (*n* = 99) (95% CI), %	After matching (*n* = 56) (95% CI), %
WT *EZH2*	35 (22–48)	50 (31–69)
MT *EZH2*	69 (55–83)	71 (54–88)

Abbreviations: CI: confidence interval; MT: mutant type, WT: wild type.

**Figure 2:**
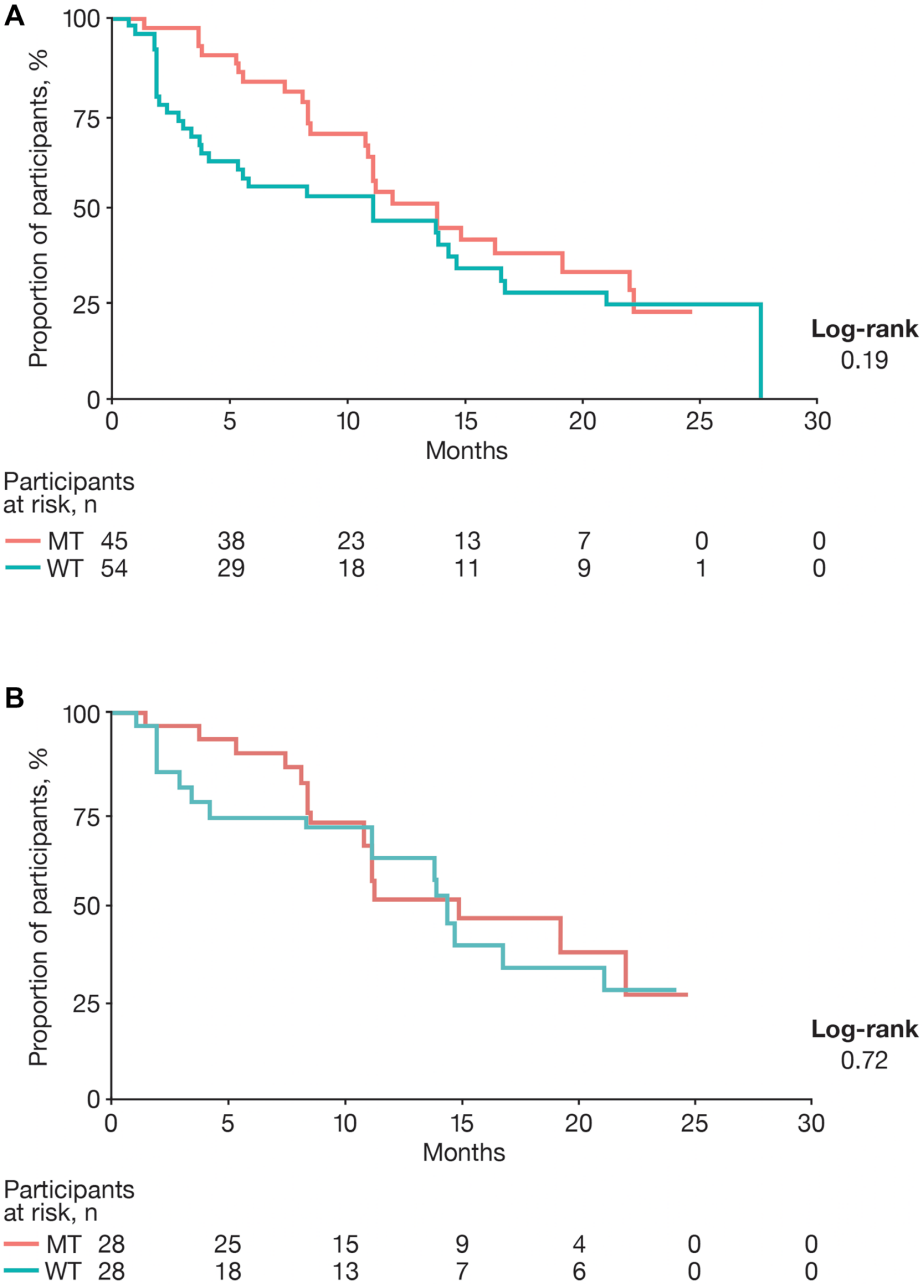
Progression-free survival. (**A**) Unmatched and (**B**) matched progression-free survival. Abbreviations: MT: mutant type; WT: wild type.

Median progression-free survival (PFS) curves and values for the 2 cohorts are shown in Figure 2 and Table 3, respectively. Prior to matching, Kaplan–Meier analyses demonstrated little overlap between PFS curves over time. Before matching, median PFS (mPFS) values were 13.8 and 11.1 months for the MT and WT cohorts, respectively. After matching, greater overlap was observed in the Kaplan–Meier curves for the 2 cohorts. Median PFS values were 14.8 and 14.3 months for the MT and WT cohorts, respectively. *P* values from the log-rank test increased from 0.19 before matching to 0.72 for the matched sample.

**Table 3:** Progression-free survival before matching

Population	Before matching (*n* = 99)		Matched sample (*n* = 56)	
	Median (95% CI), mo	Median follow-up, mo	Median (95% CI), mo	Median follow-up, mo
All participants	11.9 (10.9–16.3)	—	14.3 (11.1–22.0)	—
MT *EZH2*	13.8 (11.1–22.1)	17.9	14.8 (10.7–∞)	19.2
WT *EZH2*	11.1 (5.4–16.7)	24.1	14.3 (11.1–∞)	24.4

Abbreviations: CI: confidence interval; MT: mutant type; WT: wild type.

## DISCUSSION

The MT and WT cohorts in the E7438-G000-101 trial enrolled patients with R/R FL by *EZH2* status. In the initial analysis of the study, the higher response rate and longer PFS in the MT population suggested preferential activity related to the existence of the *EZH2* mutation. However, because the study was not designed to compare the 2 cohorts, population baseline characteristics were imbalanced between the 2 recruited cohorts. Results of the trial demonstrated efficacy of tazemetostat in both groups, with a higher rate of response seen in the MT group. However, those in the MT group also appeared to have, on average, a better prognosis. Some of this efficacy difference between the 2 groups appears to be in part due to differential (ie, imbalanced) enrollment into the trial.

Propensity score–based matching approaches can be used to reduce selection bias in the measurement of outcomes in retrospective analyses of clinical studies and provide more robust evidence of treatment effect. These techniques have often been applied to assess clinical outcomes between different intervention-based cohorts of participants across administrative claims, patient registries, and other health care databases [5, 6]. This study used individual patient data from both the *EZH2* MT and WT subgroups to create a subset of participants in each cohort matched on baseline characteristics via the propensity score. Several strengths are associated with the propensity score–based matching approach. It simulates a randomized control trial design by using statistical methods to create a matched sample of 2 groups rather than through randomization and thereby reduces confounding by mutation status through minimizing differences between baseline factors. Hence, data from dissimilar participants in the overall MT and WT populations were discarded from the sample for outcome comparison.

The resulting samples in the model were more balanced across key prognostic factors. The balancing step was followed by assessment of efficacy, in which the point estimate of the ORR for the WT sample increased from 35% to 50%, whereas the ORR for the MT group was relatively unchanged, going from 69% to 71% before and after matching, respectively. Although some differences were still observed between the cohorts in ORRs, mPFS values were very close in the sample after matching. The mPFS for the WT cohort increased from 11.1 to 14.2 months compared with 14.6 months from the MT cohort after matching, although the median follow-up time continued to be several months longer for the WT cohort, a finding that can confound such an analysis.

The results of this analysis provide further indication that tazemetostat, despite being an EZH2 inhibitor, is often effective in patients with WT *EZH2*, even if its response rate still appears higher in patients with MT *EZH2*. The mechanism of tazemetostat in patients with WT *EZH2* is not completely understood. However, most patients with FL have at least 2 mutations in chromatin-modifying proteins that can serve to prevent B cells from exiting the germinal center, making it easier for the EZH2 protein to maintain transcriptional repression unchecked, and, in such cases, the tumor can be susceptible to *EZH2* repression [4, 7–10]. Furthermore, amplification of *EZH2* is observed in 15% of people with FL, independent of *EZH2* mutation status [11]. Thus, the mechanism of action for tazemetostat in targeting EZH2 may be effective in patients without a mutation in the *EZH2* gene itself owing to other mutations that activate EZH2 activity.

Certain limitations are associated with this study. The small sample sizes of participants with MT or WT enrolled in the E7438-G000-101 trial limits the performance of the matching algorithm, which makes estimation of statistical significance difficult, and restricts the number of variables included in the model to avoid over-parameterization, which increases the risk of meaningful bias remaining. Even after the matching procedure is refined and finalized, participants in the 2 cohorts continue to remain slightly different in their matching variables. Another limitation common to this type of analysis is that baseline variables included in the propensity-estimation model were limited to those in which individual patient data were available. Therefore, other sources of variability between participants that are unmeasured or unpublished cannot be accounted for using this method or other statistical adjustment techniques.

This analysis indicates that differences in outcomes between the 2 mutation subgroups in the trial were at least partly due to differences in prognosis of the 2 populations at baseline, leading to the hypothesis that the actual difference in the effectiveness of tazemetostat by *EZH2* status may be much smaller than suggested in the headline trial results [4]. This hypothesis should be further investigated through additional clinical trials or further analysis of real-world outcomes.

## MATERIALS AND METHODS

### Data source

The tazemetostat E7438-G000-101 trial for R/R B-cell NHL and advanced solid tumors was carried out in 2 phases, with the phase 2 segment being the focus and pivotal study of this set of comparative analyses. The primary endpoint was ORR per the 2007 International Working Group NHL response criteria. Data from the FL cohorts (cohorts 4 and 5) were published in November 2020 [4]. Cohort 4 enrolled patients with FL and MT *EZH2*, whereas cohort 5 enrolled patients with FL and WT *EZH2*. The latest data cutoff dates from the FL cohorts for efficacy and safety were August 9, 2019, and May 24, 2019, respectively. The analysis described as follows relied on data from this trial in cohorts 4 and 5 alone. Individual patient data were anonymized.

### Propensity score–matching analysis

#### Overview of the propensity score–based matching analysis

A propensity score–matching approach was employed to create a comparable sample of participants between the MT and WT subpopulations. Propensity scores were generated using a logistic regression model in which the propensity score was defined as the conditional probability of receiving the treatment (in this case, having the *EZH2* mutation) given the set of observed covariates used in the model.

#### Assessing differences in cohorts and selecting covariates for matching

An initial comparison was made between the *EZH2* MT and WT subpopulations by using standardized differences for continuous and categoric variables. After a review of availability and definitions of the listed variables and reported summary statistics, covariates were selected on the basis of variables determined to be predictive or prognostic in relation to R/R FL outcomes (based on clinical opinion) and that meaningfully differed between the *EZH2* subpopulations at baseline. Considerations for selecting a shortlist of variables to include in this step involved a tradeoff between inclusion of all factors deemed to be prognostic and preventing overspecification of the estimation model, particularly given the relatively small sample size. The final variables for inclusion in the model were based on optimization for balance after matching, as described in the following section.

#### Matching participants

A nearest-neighbor approach was used to determine participants considered to be close matches based on the derived propensity scores. The threshold (caliper) for determination of the nearest neighbor was assessed based on iterative testing, model performance, and resulting balance of the matched participants. The choice of matching participants in a 1:1 or 1:many manner was determined using a similar approach.

Multiple model specifications were tested for optimization through inclusion of different combinations of baseline covariates, as well as testing optimal caliper, and 1:1 or 1:many matching. The optimal model for matching participants with *EZH2* MT or WT from the full sample using the calculated propensity scores was optimized for developing balance across the resulting sample while retaining a sufficient number of participants for analysis and sufficient coverage of key published variables. Covariates identified for inclusion in the final propensity score estimation model were Eastern Cooperative Oncology Group performance status, progression of disease within 24 months, prior autologous stem cell transplantation, number of prior lines of systemic anticancer therapy, and double refractory status. The final model used a 0.2 caliper and a 1:1 match. Data that were missing or unavailable were not included in generating estimates from the propensity score–based modeling approach.

After the matched sample of subpopulations was created, baseline characteristics in the 2 new cohorts were calculated and compared using standardized differences for continuous and categoric variables.

#### Measurement of outcomes

Comparative analyses between the *EZH2* MT and WT cohorts were conducted before and after matching. Before and after matching, ORRs (a binary outcome) were summarized. Median PFS values were described before and after matching for both cohorts using Kaplan–Meier analyses. Log-rank test was used to test for significance in the difference in PFS curves between the 2 cohorts.

## Abbreviations

ASCT: autologous stem cell transplantation
CI: confidence interval
ECOG PS: Eastern Cooperative Oncology Group performance status
EZH2: enhancer of zeste homolog 2
FDA: US Food and Drug Administration
FL: follicular lymphoma
mPFS: median progression-free survival
MT: mutant type
NHL: non-Hodgkin lymphoma
ORR: objective response rate
PFS: progression-free survival
POD24: progression of disease ≤24 months
R/R: relapsed or refractory
SD: standard deviation
WT: wild type
